# Estimating Components and Costs of Standard Care for Children with Autism Spectrum Disorder in Europe from a Large International Sample

**DOI:** 10.3390/brainsci11030340

**Published:** 2021-03-07

**Authors:** Łucja Bieleninik, Christian Gold

**Affiliations:** 1 GAMUT—The Grieg Academy Music Therapy Research Centre, NORCE Norwegian Research Centre AS, 5838 Bergen, Norway; chgo@norceresearch.no or; 2 Institute of Psychology, University of Gdańsk, 80-309 Gdansk, Poland; 3 Faculty of Psychology, University of Vienna, 1010 Vienna, Austria

**Keywords:** autism, services, direct costs, indirect costs, financial burden, health economics

## Abstract

(1) Background: European guidelines provide recommendations for services and care for people with autism spectrum disorder (ASD), but not all interventions are generally available. Knowledge of service use and costs and wider societal costs in Europe is limited; (2) Method: Using an international sample, we analysed services and costs in 357 children (4–6.99 years) with ASD based on parent reports. Costs were transformed into EU-28 average using purchasing power parity; (3) Results: 122 children (34%) received specialist autism services; 149 (42%) received sensory/motor therapy; 205 (57%) received speech/language therapy; 35 (10%) received play therapy; 55 (15%) received behavioural interventions; 31 (9%) received social skills training; 47 (13%) participated in therapeutic recreational activities; and 59 (17%) received other services. The total number of hours for these services combined over two months was M = 34 (SD = 63; range: 0 –372). Estimated total costs of health-related services were M = 1210 EUR (SD = 2160 EUR); indirect societal costs were M = 1624 EUR (SD = 1317 EUR). Regression analyses suggested that costs rise with age and presence of intellectual disabilities, but not with severity of autism; (4) Conclusions: The high extent of community-based services indicates good accessibility but also considerable variation in the receipt of services. The costs of autism services are considerable. Further research is needed to investigate whether services received match individual needs.

## 1. Introduction

Autism spectrum disorder (ASD) refers to a range of lifelong neurodevelopmental conditions characterized by the presence of early appearing symptoms in two core domains: impairments in reciprocal social communication and the presence of restricted interests and repetitive behaviours (DSM-5; ICD-11) [1,2]. ASD affects about 1% of population with a high variability of estimates worldwide [3], with a rising trend [4], and is around 3–4-times more frequent in males than in females [5,6]. Most of the behavioural, cognitive and socio-emotional features of ASD are associated with genetic, biological, environmental, and developmental influences [7,8,9].

### 1.1. Recommended Interventions for Children with ASD in Europe

It is important to provide individuals with ASD and their families with support to live their best possible life. There is no cure for autism nor one care package that suits everyone. People with autism require different support at each age of their lives, and it is therefore essential to ensure the right type of evidence-based therapy at the right time. Relevant guidelines often emphasize general principles in the absence of more specific evidence. One relevant policy overview by World Health Organization (https://www.who.int/news-room/fact-sheets/detail/autism-spectrum-disorders, accessed on 1 April 2020) recommend evidence-based psychosocial interventions including behavioural treatment and parent skills training programmes which can minimalize limitation in communication skills and social behavioral skills with improving well-being and quality of life for both individuals with ASD and their parents. Based on current best evidence, guidelines developed by Autism-Europe [10] recommend four fundamental principles of good practice: individualisation (diversity, personalised support); structure (predictability, stability); intensity and generalization (not sporadic or short-term, but systematic and across settings); and family participation (valuing parents’ role and respecting family values and culture). A variety of approaches have been developed to improve the core deficits of ASD, with the strongest evidence with behaviorally based approaches. However, approaches that are commonly used are those which aim to improve parent-child interactions and improve social and communication skills. Since core symptoms of ASD are remarkably stable over time across childhood, intervention studies should prioritize the improvement of well-being, quality of life and adaptive functioning [10]. Autism-Europe also highlights the importance of identifying what a good quality of life means for individuals with ASD, since their needs may differ from the typical population [11].

### 1.2. Costs of Standard Care for Individuals with ASD Across the Lifespan

ASD is often associated with poorer quality of life of individuals and their families. While some individuals with ASD are able to lead productive lives, others suffer lifelong loss of economic productivity and have long-term complex needs for health and social care services, special education, leisure services, and housing placements [12,13,14]. The cost of ASD services is generally high; it increases with the presence of intellectual disability and varies across age groups [13,15,16,17]. In addition to costs of services, substantial economic burden is linked with lost parental income due to increased demands on the primary caregiver. In particular, mothers of autistic children work not only fewer hours per week, but also earn less than mothers of healthy children or mothers of children with other disabilities or disorders [13].

Most studies on economic costs have been conducted in the US [13,14,17,18,19,20,21,22,23,24,25], while some others in the UK [13,15,16], Sweden [26], the Netherlands [27], Ireland [28], China [29], Australia [30], Canada [31]. Thus, it remains unclear what services are available for individuals with ASD and what costs of services received by individuals with ASD are in Europe. This study aims to contribute to this growing area of research by exploring data from 5 European countries and 4 non-European countries. A primary objective of this study was to investigate services received by children with ASD and associated costs in Europe. The secondary purpose of this investigation was to explore the relationship between child’s age, symptom severity, intellectual disability and costs of services as well as to compare data for European and non-European countries.

## 2. Materials and Methods

### 2.1. Study Design and Participants

Using the dataset of the multinational randomised controlled Trial of Improvisational Music therapy’s Effectiveness for children with Autism (TIME-A) [32], we analysed the services received and associated costs. Of an original sample of 364 children, 357 had complete baseline data and were used for this study. Data were available for 217 children from 5 European countries (Austria, Israel, Italy, Norway, UK) and additionally for 140 children from non-European countries (Australia, Brazil, Korea, USA). Participants were assessed between 2011 and 2015. Eligible children were aged 4–6.99 years and diagnosed with an ASD (ICD-10 codes: F84.0; F84.1; F84.5; F84.9) based on the Autism Diagnostic Observation Schedule (ADOS) and Autism Diagnostic Interview–Revised (ADI-R). The children’s level of cognitive ability was assessed using the Kaufman Assessment Battery for Children (K-ABC) [33] or another standardised IQ test. In case the child was unable to complete a formal test, clinical judgment was used. Except serious sensory disorders, all comorbidities were allowed. According to the TIME-A protocol [34] children who had received music therapy during the last year were excluded.

Details on study procedures were described separately [34], while a flow chart through recruitment and results can be found in Bieleninik et al. [32]. Ethics approval was obtained by the relevant ethics committees or institutional review boards at each site. Freely given, written informed consent was obtained from each participant’s parents/caregivers before any study procedures occurred.

### 2.2. Data Collected and Management

TIME-A used assessments by blinded clinicians as well as reports by parents/caregivers collected at several time points. For the current study purpose, only data collected at baseline on scores of the ADOS and ADI-R, IQ level, as well as costs and concomitant treatments were included. Parents answered a detailed questionnaire regarding services received and out-of-pocket expenses during the past two months. The following data were collected:

1. Type of therapy or intervention (individual vs. group settings; number of sessions; average duration of one session; private costs per session);
2. Institutional stay (number of admissions; number of days spent in facility; kind of facility/institution; private costs incurred);
3. Outpatient treatment (number of visits of psychiatric/somatic services; number of visits of other specialized services; private costs incurred);
4. Dietary supplement and medication (name of the drug/supplement, dosage, duration, private costs incurred);
5. Special diet (kind; estimated extra cost);
6. Care giver investment:
  - Working hours spent on treatment as described above, including driving, of all private care givers (weekly average in hours);
  - Work situation (working or not working);
  - Reduced hours due to the child’s needs (in percentage);
  - Employing anybody privately to help care for the child (if yes, private costs incurred);
  - Any additional assistant/aide in school/at home for the child (if yes, number of hours per week);

Respondents were asked to reply with regard to the last 2 months. The limited recall time frame was used to minimalize the recall bias following other economic evaluations e.g., [35,36]. The 2 months recall frame is the appropriate period of the recollections retrieved by parents/caregivers in terms of retrospective estimations of costs bear by them. The questionnaires were completed on paper and double-entered manually into the system to ensure data quality. All data were stored on an electronic database management system located on a secure server with password-controlled access (OpenClinica software for clinical research, version 3.3).

### 2.3. Assessment of Costs

Unit costs were inserted where a service was used but parents reported no expense for the service. Costs were transformed into EU-28 average using purchasing power parity (https://data.oecd.org/conversion/purchasing-power-parities-ppp.htm, for 2018, accessed on 4 March 2021). All health services costs (including special autism services, sensory-motor therapy, speech language therapy, behavioural intervention, social skills training, therapeutic recreational activities, other) were sourced from personal communication and represent typical costs in Austria, a Central European country and one of the countries where data were collected. Heath services costs were set up to be 84 EUR per 60 min (one session) based on 1:1 setting, and discounted for group settings, assuming a group size of 5. Costs of institutional stay (310.08 EUR per day) and outpatient treatment (167.10 EUR per contact/visit) were retrieved from a report of Unit Costs of Health and Social Care [37]. Societal costs regarding childcare, including data on full-time child care (353.79 EUR per month), part-time child care (181.73 EUR per month), and schooling (211.74 EUR per month), were sourced from the 19th Annual Childcare Survey [38], again transformed using purchasing power parity. Productivity losses were calculated using the human capital approach. The education level of both parents and the percentage of reduced working hours, as reported by parents, was used. According to the EU statistics on income and living conditions (EU-SILC, https://ec.europa.eu/eurostat/en/web/products-datasets/-/ILC_DI08, accessed on 4 March 2021), the following unit costs were obtained: 1946.08 EUR per month for full-time work where both parents had a university degree; 1391.42 EUR per month where both parents had 12 or more years of education; and 1129.42 EUR per month where at least one parent had less than 12 years of education or education level was unknown. No unit costs were imputed for medication and special diets because these costs were found to be negligible in children with ASD [15]. We did not adjust for inflation, because the annual inflation rate from 2013 to 2017 (the years the study data were collected) was found to be negligible (https://ec.europa.eu/eurostat/tgm/table.do?tab=table&init=1&language=en&pcode=tec00118&plugin=1, accessed on 4 March 2021).

### 2.4. Statistical Analyses

The main analyses were based on the entire dataset. Means, SDs, ranges, and frequencies were calculated for each type of service as applicable and presented in tables. Costs were presented in the same way, along with sums of all health services costs, indirect societal costs, and sum of all costs.

#### 2.4.1. Regression Analysis

A regression analysis was used for the estimation of relationship between a child’s age, severity of autism symptoms (measured with ADOS total algorithm score), and intellectual disability (below-average intelligence) and health services costs and societal costs. Following literature that the age and severity of autism symptoms are significant predictors of costs for children at preschool age [15] and in adolescents [39] we expected the health services costs and societal costs to vary with the child’s age and symptom severity. A large body of research has revealed that individuals with intellectual disability have higher costs e.g., [13]; thus, we predicted that health services costs and societal costs will be higher for children with intellectual disability (defined by IQ < 70) compared to those without (defined by IQ > 70). The distribution of values was examined graphically, and any clear outliers were removed.

#### 2.4.2. Sensitivity Analysis

A sensitivity analysis was used to determine how data retrieved from non-European countries affected the obtained health services costs and societal costs. In order to determine the robustness of our results by examining the extent to which results of health services costs and societal costs are affected by country of origin, we calculated costs including only 217 children from Europe (5 European countries: Austria, Israel, Italy, Norway, UK) and excluding individuals from other non-European countries (Australia, Brazil, Korea, USA; *n* = 140).

## 3. Results

### 3.1. Participant Characteristics

The total of 357 children (83% boys) were included with a mean of age 5.4 years (SD = 0.9). As can be seen from Table 1, the majority of the children (83%) were diagnosed with a childhood autism (ICD-10 code F84.0), while only a few had types of autism, including atypical autism (F84.1; 1%); Asperger’s Syndrome (F84.5; 4%); pervasive developmental disorder unspecified (F84.9; 13%). Mean scores of the ADOS (Module 1—61% of included children; Module 2—36%, Module 3—3%) were as follows: total score 17.7 (SD = 5.3), social affect 13.8 (SD = 4.4), language and communication 3.3 (SD = 1.5), reciprocal social interaction 10.5 (SD = 3.6) and restricted and repetitive behavior 3.9 (SD = 2). Mean scores of the ADI-R were 18.2 (SD = 5.8) for reciprocal social interaction, 13 (SD = 4.2) for language and communication, 5.8 (SD = 2.4) for repetitive behaviors and interests, and 3.9 (SD = 1.1) for early onset. The IQ was evaluated by using standardised measurement tool (*n* = 217) and clinical judgement (*n* = 140), the results of which indicate that 47% of children (*n* = 163) had IQ below 70. The majority of individuals attended to school (62%), while 22% to full-time care and 11% to part-time care. More details can be found in the top half of Table 1.

In regard to parents’ situation of work, the majority of mothers were homemakers (34%) followed by 26% being part-time employed, while the majority of fathers were working full-time (74%). Regarding education, many parents had a university degree (46% of mothers; 43% of fathers), followed by education equivalent to high school (37% of mothers; 32% of fathers), and less than high school (10% of mothers; 14% of fathers). The bottom half of Table 1 shows more details about caregivers; and family characteristics.

### 3.2. Services Used

Of 357 children, 122 (34%) received specialist autism services; 149 (42%) received sensory/motor therapy; 205 (57%) received speech/language therapy; 35 (10%) received play therapy; 55 (15%) received behavioural interventions; 31 (9%) received social skills training; 47 (13%) participated in therapeutic recreational activities; and 59 (17%) received other services (Table 2). The total number of hours for these services combined over two months was M = 34 (SD = 63; range: 0 to 372). The majority (N = 222; 62%) of included children attended school, 33% (N = 118) attended kindergarten (wherein 22% full time, 11% part-time); 5% (N = 17) attended none of the aforementioned. Regarding other services, twelve (3%) had an institutional stay; 37 (10%) received outpatient treatment; 109 (31%) received any type of medication; 59 (17%) followed a special diet. It can be seen from the data in Table 2 that many parents spent time with their children’s services (N = 175; 49%); worked reduced hours (N = 191; 54%); or employed a child care help (N = 47; 13%) or an additional aide at school or home (N = 108; 30%).

### 3.3. Costs of Services

Table 3 presents health services and social costs. Estimated total costs of health-related services were M = 1210 EUR (SD = 2160 EUR). Specialist autism services; behavioural intervention; and speech/language therapy were associated with the highest costs (respectively, M = 315 EUR; M = 284 EUR; M = 251 EUR). High costs were also associated with sensory/motor therapy (incl. occupational & physiotherapy) at M = 213 EUR (SD = 460 EUR). Lower costs were observed for play therapy (M = 32 EUR; SD = 194 EUR), therapeutic recreational activities (e.g., horse-riding; M = 23 EUR; SD = 178 EUR) and social skills training (M = 12 EUR; SD = 64 EUR). However, it should be noted that other costs incurred by the parents were also considerable (M = 63 EUR; SD = 348 EUR).

The sum of indirect societal costs was M = 1624 EUR (SD = 1317 EUR; Table 3). An additional aide at school or home represented the highest costs in this category (M = 621 EUR; SD = 1146 EUR) followed by reduction in working hours (M = 524 EUR; SD = 643 EUR), childcare/school (M = 461 EUR; SD = 160 EUR), and child care help (M = 18 EUR; SD = 125 EUR).

Total costs including health services costs and societal costs were estimated to be around 2834 EUR (SD = 2498 EUR) over the last 2 months with a wide range (84 EUR to 26,051 EUR). It can be seen that the SDs are relatively high compared to the means; sometimes higher than the means. This reflects the skewed nature of these data, which is a common phenomenon in health economic data [40,41].

### 3.4. Regression Analysis

**Figure 1:**
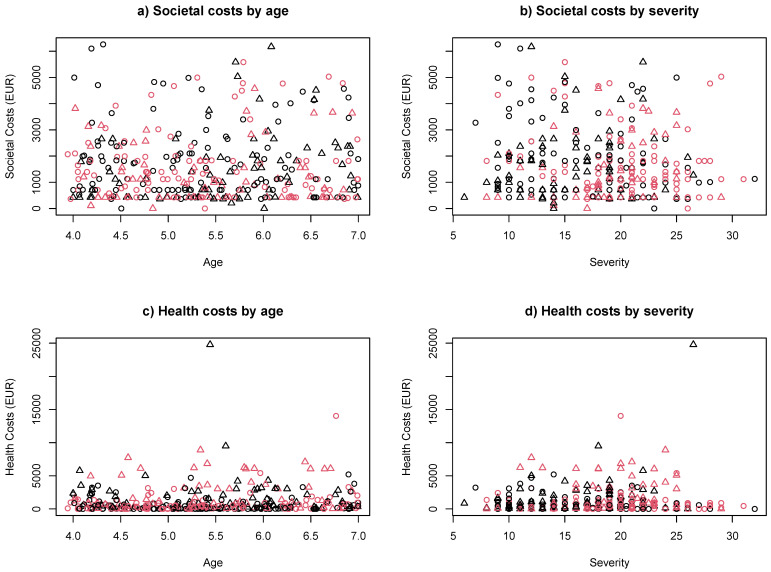
Results of regression analyses. (**a**) Societal costs by age; (**b**) Societal costs by severity; (**c**) Health costs by age; (**d**) Health costs by severity. Severity was measured using ADOS Total scores (adapted as described in Bieleninik [32]. Red—intellectual disability; black—no intellectual disability or unknown (8 cases). Circle—in Europe (including Israel); triangle—outside Europe.

Table 4 provides the results obtained from the regression analysis of health services costs and societal costs. In the analysis with all participants, children’s age, severity of autism symptoms, intellectual disability, and the health costs and societal costs did not show significant influence on any variable. However, standard errors were large and the estimates did not exclude potential relevant effects. Additionally, the scatterplots in Figure 1 (panels c, d) showed one outlier whose health costs were about twice as high as all other values. Thus, we re-ran the regression analyses without this outlier in the data. This analysis suggested a significant effect of children’s age (*p* < 0.048) and intellectual disability (*p* < 0.005) on health costs (Table 4A, middle column). These findings suggest that total health costs increase with higher age and with IQ below 70. There was no relationship between autism severity measured with ADOS in any of these analyses. The results indicated that health costs increased by 213 EUR with each additional year of age and by 566 EUR when children also had an intellectual disability.

Figure 1 also illustrates the relation between these predictors and health and societal costs. In general, this Figure shows a high diversity of costs across all levels of intellectual functioning (indicated by black/red colour) and place of living (Europe vs. outside Europe, indicated as circle/triangle) in the whole range of children’s age (panels a, c) and symptom severity (panels b, d). The diversity is higher for societal costs (panels a, b), with cost values distributed more evenly across the whole range, than for health costs, where there are many low values and fewer cases with much higher costs. This pattern is common for health costs [40,41]. While families spent between 0 EUR and 5000 EUR on health services, a few spent up to 10,000 EUR or more on their child.

Considering the societal costs, there is one pattern to observe (Figure 1a,b). The highest costs are observed in those with intellectual disability (shown in red), and those with intellectual disability also show the highest severity of symptoms (expressed with higher ADOS total scores). Importantly, children with intellectual disability were represented in all age groups within the sample. In total, while societal costs ranged from 0 to more than 6000 EUR, the majority of families had costs between 0 and 3000 EUR.

### 3.5. Sensitivity Analysis

The results obtained from the sensitivity analysis has shown that for individuals from European counties the pattern of services used did not differ much from non-European countries. Of 217 children (Table 5), 50 (14%) received specialist autism services; 72 (20%) received sensory/motor therapy; 109 (31%) received speech/language therapy; 21 (6%) received play therapy; 19 (5%) received behavioural interventions; 15 (4%) received social skills training; 25 (7%) participated in therapeutic recreational activities; and 25 (7%) received other services. Details on the total number of hours for health services combined and number of sessions over two months are presented in Table 5.

The results about schooling indicate that the majority of individuals from European counties (*n* = 121; 34%) attended school, 91 of included children (25%) attended kindergarten (wherein 18% full time, 7% part-time); while 5 (1%) attended none of aforementioned. Regarding other services, 10 children (3%) had an institutional stay over the last 2 months; 24 (7%) received outpatient treatment; 55 (15%) received medication; and 38 (11%) a special diet. It can be seen from the data in Table 5 that many parents spent time with their children during health services used (*n* = 85; 24%); 111 had reduction in working hours (31%) due to a child care assist (*n* = 26; 7%) and required an additional aide at school or home (*n* = 77; 22%).

Table 6 presents health services and social costs for individuals from European counties. Estimated total costs of health-related services were M = 740 EUR (SD = 1323 EUR) with a rage from 0 EUR to 14,042 EUR. Speech/language therapy; behavioural intervention; and sensory/motor therapy (incl. occupational & physiotherapy) were associated with the highest costs (respectively, M = 201 EUR; M = 187 EUR; M = 123 EUR). High costs were also associated with specialist autism services at M = 98 EUR. Lower costs were observed for play therapy (M = 48 EUR), other services (M = 53 EUR), and social skills training (M = 14 EUR). The sum of indirect societal costs was M = 1748 EUR (SD = 1345 EUR). Additional aide at school or home was linked with the highest of all societal costs (M = 753 EUR; SD = 1221 EUR) followed by childcare/school (M = 492 EUR; SD = 156 EUR), reduction in working hours (M = 487 EUR; SD = 590 EUR) and child care help (M = 17 EUR; SD = 111 EUR). Total costs including health services costs and societal costs, were estimated to be around 2489 EUR (SD = 1951 EUR) over last 2 months with a wide range (101 EUR to 15,860 EUR).

## 4. Discussion

### 4.1. Main Finding

Previous studies evaluating services and costs observed consistent results about a substantial economic burden of ASD [13,15]; however, most studies have paid particular attention to the UK and the US. Recently, there has been renewed interest in the economic costs associated with ASD e.g., [28,42], but the services and costs were not fully presented in Europe yet. The current study, which was based on an international sample of young children, confirmed that the costs of autism services are considerable. The total costs for children with autism including health services costs and societal costs were estimated to be around 2834 EUR over a period of 2 months. The costs of health-related services were around 1210 EUR and indirect societal costs were 1624 EUR. Health services costs rose by 213 EUR per additional year of child’s age and by 566 EUR for children with intellectual disability. The pattern of services used is similar (but not the same) for children from European countries and non-European countries addressing particularly to differences in using behavioral treatment. Among health-related services the specialist autism services (315 EUR), behavioural intervention (284 EUR), and speech/language therapy (251 EUR) were key cost drivers, while among indirect societal costs the employment of additional aide at school or home was key cost driver. To our knowledge, this study present, for the first time, data based on an international cohort in both European and non-European countries.

### 4.2. Health-Care Service Used

The results of our study indicated that among all types of services, speech and language therapy was most often used by young children with ASD (57%). It may be explained by the fact that communication problems belong to the core autism symptoms; thus, children may benefit the most from this kind of treatment at this early stage of life. The second and most frequently chosen form of therapy was sensory and motor therapy (including occupational therapy and physiotherapy). High use (42%) of this intervention might be due to the core nature of sensory symptoms in individuals with ASD, particularly to sensory over-responsivity [43]. On the other hand, motor deficits belong to disturbances supporting the diagnosis of ASD according to DSM-5 and they have been showed as moderators of autism core symptoms [44]. Around 34% of the included children received specialist autism services, including special education schools specifically for children with ASD. This result may be explained by the fact that approx. 47% of them had intellectual disabilities. Children with ASD are in broad range of diversity in cognitive ability from intellectual disability to average or superior intelligence. While some children with autism use regular school services, a greater proportion within this group of children require special education services [14] which impact on higher societal costs. 13% from our cohort received therapeutic recreational activities, 10% play therapy, and 9% social skills training. Targeting to the aforementioned kind of interventions is a response to the presence of the impairment of social interaction which belongs to the core autism symptomatology. The most surprising aspect of the services used is that behaviour intervention (e.g., treatment and education of autistic and related communication handicapped children—TEACCH; applied behavioral analysis—ABA) was chosen only by 15% of the included children. Even though evidence supports early intensive behavioural intervention (EIBI)—programmes for some children with ASD [45], the low percentage of children using behaviour intervention in our study might be due to the high cost [27,46], long duration and high intensity. Findings from our cohort reflect the diversity of approaches recommended in guidelines worldwide. The high extent of community-based services indicates good accessibility but also considerable variation in the receipt of these services.

### 4.3. Costs of Health Care Services

Estimated total costs of health-related services were M = 1210 EUR for a 2-month period, while specialist autism services contributed to these costs the most (315 EUR) followed by behavioural intervention (284 EUR) and speech/language therapy (251 EUR).

Roggie and Janssen [46] in their systematic review found that EIBI is the most common form of therapy delivered to children with ASD; however, there is a limited amount of research which estimated the costs of this service. The annual costs for behavioural intervention has been estimated to be from USD 40,000 [47], USD 45,000 [48], USD 50,000 [49] even exceeding USD 60,000 [50] for American cohorts. Pilot research conducted in UK on 10 children participating in EIBI [51] found that weakly costs of this service were about GBP 144, of which 33% of costs are borne by the parents. Data based on 33 children with ASD from Sweden indicated that annual average cost oscillated around 4088 EUR [52]. Another study conducted in the Netherlands on individuals with ASD from age 3 to 65 years [27] showed that total EIBI programme costs are around EUR 100 000 for 33 h per week across 27 months. Tsiplova and colleagues [42] evaluated resource utilization and costs for 263 children with ASD in two Canadian provinces during one year. Even though variation in the EIBI cost per child was observed across the two provinces, the public high costs incurred were mainly related to EIBI costs (from CAD 29,975 to CAD 90,882 per child). In their research, families had relatively low out-of-pocket costs per child before starting EIBI (CAD 3046) compared to CAD 3713 during EIBI (mostly due to transportation expenses), while the opposite pattern was observed for the second province (a decrease from CAD 2560 to CAD 1130) [42]. Even though EIBI programmes are associated with high costs, often from private family resources, participating in those services is linked with significant cost savings over the life span [27,47,48,50,53]. Analyses done by Lavelle and colleagues [14] on national data from the Medical Expenditure Panel Survey including children from 3 to 17 years indicated that annual mean costs of ASD-related therapies and other family coordinated services are CAD 350. However, there is a limited number of analyses of costs related to other forms of therapy in the literature. One study by Byford and colleagues [54] analysing severity of autism symptoms of 52 children with autism (aged 2–4;11) randomly allocated to treatment as usual (TAU) or a parent-mediated, communication-focused therapy (PACT) combined with TAU, observed improvement for group PACT and TAU compared with TAU (53% vs. 41%, respectively). Their results indicated that the total cost of the PACT was GBP 4105 per child with an average cost of one PACT session to be GBP 264. Total costs of health, education and social service were calculated on GBP 6539 per child in the treatment group and on GBP 2050 in comparison group [54].

### 4.4. Costs of Other Health Care Services

The present results indicated a high diversity among children’s needs and therefore health care services used. Institutional admissions occurred in 12 cases (3% of cohort), while visits to psychiatric, somatic, or other specialized outpatient services occurred in 37 cases (10%). Even though our data indicated a relatively low mean cost of institutional stay (3 EUR) and outpatient services (1 EUR) over a period of two months when averaging across the whole sample, the wide range of costs show that there were families who incurred very high costs (981 EUR and 132 EUR, respectively) related to these services, whereas other families did not have these costs at all. A large and growing body of literature has investigated healthcare utilization expenditures for individuals with autism. Other research in medical and healthcare costs has emphasized the higher costs for individuals with autism compared to individuals without [14,19,21,22] and with other mental health problems [23]. One of the reasons for this difference is that people with autism use such services more often than the general population [46]. Some studies also reported increasing medical and healthcare-related costs over the lifetime [18]. For example, Cidav [55] found that the largest increase in inpatient care takes place between 3–6 and 7–11 of age, for outpatient care between 7–11 and 12–16. Ganz [22] found that inpatient costs were 2.9 times while outpatient costs were 2.5 times greater for children with autism compared to children without ASD. Another study showed also higher costs for adults with ASD than for children with ASD [13]. Rogge and Janssen [46] in a review indicated a number of reasons why the results concerning these costs should be cautiously interpreted.

One third (31%) of the included families in our sample experienced some costs related to medication. Even though the present data indicate a relatively low cost of medication (approx. 6 EUR) over a period of two months, the range of costs (from 0 to 290 EUR) emphasizes that there were families who incurred very high costs related to these services, and families who did not have these costs at all. There is a large volume of published studies e.g., [19] describing the high costs related with ASD to health care utilization. Data in literature indicate that medical expenditures were around 6 [17,18], 7.6 [22], and even 10 times [20] greater for children with ASD compared to children without ASD. Research by Järbrink and Knapp [26] suggested that costs for medication contributed to 72.6% of the total costs for autistic children with additional disability in comparison with 39.8% for high-functioning children with autism. Another research by Croen and colleagues [19] including 3053 children (2–18 years of age) with ASD and 30 529 without, suggested that annual costs for hospitalizations, clinic visits, and prescription medications were two times higher in those with autism (respectively: CAD 550 vs. CAD 208; CAD 1373 vs. CAD 540; CAD 724 vs. CAD 96). In their study, 3% children with ASD experienced inpatient and 5% outpatient hospitalizations compared to 1% and 2% (respectively) for children outside this group.

Seventeen percent of the included children in our study experienced costs associated with a special diet; across all included children, the cost of special diet was on average 7 EUR over a period of two months. The broad range of these costs from 0 to 455 EUR indicates that some families have had a very high cost associated with a special diet. These results are likely to be related to a possible relationship between nutritional status and general condition of ASD children. Peretti and colleagues [56] emphasizes that gluten-free, casein-free and ketogenic diets as well as vitamin D and folic acid improve social, cognitive, motor, and communicative skills of children with autism.

### 4.5. Costs of Indirect Societal Costs

Results of the present study indicated the indirect societal costs to be around 1624 EUR over a period of two months. In terms of societal costs, an additional aide at school or home contributed the highest costs (621 EUR), followed by reduction in working hours (M = 524 EUR), childcare/school (M = 461 EUR), and child care help (M = 18 EUR).

Our findings seem to be consistent with other research which found a high economic burden linked with ASD on society [46]. For instance, education costs constituted approximately 90% of the total costs for adolescents [39]. Even though some estimation indicates that special education costs declined with age [22,39], they are one of a major contributor to costs for parents with children with ASD [14]. Järbrink and Knapp [26] emphasize the dependence of costs on the functioning of the person, e.g., special education costs constitute 6.1% of the lifetime cost for autistic person with additional learning disability, while 13.8% for high-functioning person with autism.

Families of individuals with ASD face significant economic burden due to reduction or interruption of employment [46]. The results of our study showed that 54% of parents had reduced their working hours due to the child’s needs, 49% of parents spent time on services with their child (including driving, and all private care givers). Only 13% of parents employed somebody privately to help care for their child, while 30% of the children needed an additional assistant/aide in school/at home. Our data has confirmed that having a child with autism is related to caregiver time investment and lost employment opportunities, which is consistent with previous findings [13,22,57]. Our view is supported by another research. For instance, mothers of autistic children are 6% more likely be unemployed, work 7 h less per week [55] and earn less compared to mothers of healthy children (56%) and mothers of children with other disabilities or disorders (35%) [13]. In addition, another study has shown that individuals with ASD where both parents/caregivers are working are as rare as 9% [55]. Having child with an autism affects family experiences, where caregivers dedicate time and on providing a suitable therapy for the child. At the same time, the reduction in working time can be choose deliberately to ensure better quality of family live, tightening the emotional bond with family members and make life meaningful.

Data from our study indicate that the reduction in working hours was linked with costs of 524 EUR; child care help with additional costs of 18 EUR over a period of two months. Our findings have confirmed previous results [13,30] that having a child with autism is related to caregiver lost work productivity and earning and is one of the biggest contributors to total societal costs. Another study revelated that the lost productivity constituted approximately 89% of the total family cost [30], while costs associated with caring for children with ASD were estimated to be around CAD 17,000 per child annually [14]. There are also reports indicating that lost productivity by parents of children with ASD and care contribute to a small portion of the overall total lifetime cost [15,26,52]. However, research by Järbrink and Knapp [26] indicated that cost for living support contributed to 72.6% of the total costs for autistic children with additional ability in comparison with 39.8% for high-functioning children with autism.

### 4.6. Regression Analysis of the Predictors of Costs

It was hypothesized that younger participants with a diagnosis of autism with intellectual disability will bear higher costs compared to those with older age, with broader ASD category and with intellectual disability. Regression calculation of costs confirmed that health costs change with age for children with ASD during ages 4–7 and with presence of intellectual disabilities, but not with societal costs. Our results indicated that health costs increased by 213 EUR per additional year of child’s age and by 566 EUR for children with intellectual disability, indicating that both age and intellectual disability are significant factors related to total health costs. However, such a relationship was not observed for the social costs.

Our results are in line with earlier findings. Research by Järbrink [52] found that age is a factor which drives the increase in expenditures by 5% with each year of age between 3 and 20 years of age with a tendency to higher costs for smaller children. Another study by Barrett and colleagues [15] also revelated that children with older age have significantly higher service costs. Buescher and colleagues [13], based on cohort from US and UK, found that mean annual costs for a child with intellectual disability were higher than those without.

The results of our study did not confirm the predictive role of symptoms severity in the health costs and societal costs measured by ADOS total score, which is consistent with another study [15]. However, Barret and colleagues [15] found that total cost of services was significant associated with autism severity as measured by the ADI-R.

### 4.7. Sensitivity Analysis of Health Services and Societal Costs

Little is known about autism-related costs in Europe. Total costs including health services costs and societal costs were estimated to be around 2834 EUR for the whole sample size compared to 2489 EUR for individuals from Europe. The results obtained from the sensitivity analysis showed that for children from European countries the pattern of services used is similar (but not exactly the same) for those from non-European countries. Children outside Europe attended more often health care services in the pre-school period, which has a direct impact of health services costs (1210 EUR vs. 740 EUR for the 2 months). Speech/language therapy; behavioural intervention; and sensory/motor therapy were associated with the highest costs for children from Austria, Israel, Italy, Norway, UK; while for the whole sample the main cost drivers were specialist autism services followed by behavioural intervention and speech/language therapy. Estimated societal costs (including childcare, productivity loss, private child care help and additional aid at school or home) were relatively close both in Europe and outside (1748 EUR vs. 1624 EUR, respectively) with the same key cost driver (additional aide at school or home; 753 EUR vs. 621 EUR vs. respectively).

### 4.8. Limitation

The generalisability of these results is subject to certain limitations. For instance, the most important limitation lies in the fact that the study used self-reported data of costs threatened by self-reporting bias as: social desirability and recall bias [58], but there is no evidence to support alternative methods for this purpose which will be more precise [59]. Second, we aimed to provide total costs estimated for Europe, but data are from 5 European countries (Austria, Israel, Italy, Norway, UK) and additionally for 4 non-European countries (Australia, Brazil, Korea, USA). We attempted to address to this limitation by using sensitivity analysis which showed that the sum of all costs were similar. We aimed to achieve some comparability across Europe through the following methodological choices: data collection was based on “real resources” rather than monetary units; “translation” of out-of pocket expenses by using the purchasing power parity approach; and estimation of unit costs represent the EU as a whole. Since there are differences between European countries and non-European countries not only in economic wealth and price levels, but also qualitative differences in how health systems are organized, the results of our study should be seen as a first step towards a European estimate of ASD service use and costs. Third, economic evaluation was based on the unit costs of services which is a common approach in costing process, but provides uncertainty. Forth, the collected data are limited to participants who have been approached to the TIME-A project (related to music therapy); thus, not everyone had an equal chance of being informed about the study in participating sites. The sample may not be strictly representative of the population. However, as a pragmatic trial, the TIME-A trial was intentionally designed with generalizability in mind, with broad inclusion criteria and very limited exclusion criteria; this may strengthen our confidence in the generalizability of the findings. In sum, due to not using a probability sample, our findings have to be generalized with some caution, because they may not represent the total population.

## 5. Conclusions

The present research explores, for the first time, the costs of services received and associated costs for children with ASD bason on 5 European countries (Austria, Israel, Italy, Norway, UK) and 4 non-European countries (Australia, Brazil, Korea, USA). Total costs including health services costs and societal costs, were estimated to be around 2834 EUR over last 2 months with a wide range from 84 EUR to 26,051 EUR. Estimated total costs of health-related services were around 1210 EUR (with a wide range from 0 to 24,780 EUR) and indirect societal costs were 1624 EUR (with a wide range from 0 to 6263 EUR). Specialist autism services; behavioural intervention; and speech/language therapy were key cost driver in this group (respectively, 315 EUR; 284 EUR; 251 EUR) followed by sensory/motor therapy (213 EUR) play therapy (32 EUR), therapeutic recreational activities (23 EUR) and social skills training (12 EUR). Among indirect societal costs the important cost driver was an additional aide at school or home (621 EUR) followed by reduction in working hours (524 EUR), childcare/school (461 EUR) and child care help (18 EUR).

The current findings add to a growing body of literature on services and costs for children with ASD by confirming that families of children with ASD bear higher costs related to the child’s age (raised by 213 EUR per additional year of child’s age) and child’s intellectual functioning (raised by 566 EUR for those with intellectual disability). This study has also shown that the diversity of approaches recommended in guidelines across countries. The pattern of services used by children with ASD from European countries is similar (but not exactly the same) for those from non-European countries. The high extent of community-based services indicate good accessibility but also considerable variation in the receipt of these services. Notwithstanding the limitations, these results suggest that the costs of autism services are considerable. There is still abundant room for further progress in evaluating data on services received by individuals with ASD and associated costs for Europe. To develop a full picture of costs additional studies targeting adolescents and adults with ASD is recommended. Further research is needed also to investigate whether services received match individual needs.

## Figures and Tables

**Table 1:** Clinical and demographic characteristics of 357 children with autism spectrum disorder.

Characteristics		No.	Value
Age, mean (SD), y		357	5.4 (0.9)
Sex, No. (%)	Boys		295 (82.6)
	Girls		62 (17.4)
Diagnosis, No. (%)		357	
	Childhood autism (ICD-10 code F84.0)		295 (82.6)
	Atypical autism (ICD-10 code F84.1)		3 (0.8)
	Asperger syndrome (ICD-10 code F84.5)		14 (3.9)
	PDD (ICD-10 code F84.9)		45 (12.6)
ADOS ^1^, No. (%)		357	
	Module 1		218 (61.1)
	Module 2		128 (35.9)
	Module 3		11 (3.1)
ADOS score, mean (SD)			
	Total	356	17.7 (5.3)
	Social affect	357	13.8 (4.4)
	Language and communication	357	3.3 (1.5)
	Reciprocal social interaction	357	10.5 (3.6)
	Restricted and repetitive behavior	356	3.9 (2)
ADI-R score ^2^, mean (SD)			
	Reciprocal social interaction	357	18.2 (5.8)
	Language and communication	357	13 (4.2)
	Repetitive behaviors and interests	357	5.8 (2.4)
	Early onset	357	3.9 (1.1)
IQ source ^3^, No. (%)		357	
	KABC		8 (2.2)
	Other standardized test		209 (58.5)
	Clinical judgment		140 (39.2)
IQ, standardized test ^4,^ mean (SD)		210	75.5 (26.3)
Intellectual disability (IQ < 70), No. (%)		349	163 (46.7)
Child care, No. (%)		357	
	Attends school		222 (62.2)
	Full-time care (≥7 h/d)		80 (22.4)
	Part-time care (<7 h/d)		38 (10.6)
	None of the above		17 (4.8)
Maternal education, No. (%)		351	
	<12 y (less than high school)		35 (10)
	≥12 y (equivalent to high school)		130 (37)
	University		161 (45.9)
	Unknown		25 (7.1)
Paternal education, No. (%)		347	
	<12 y (less than high school)		50 (14.4)
	≥12 y (equivalent to high school)		112 (32.3)
	University		149 (42.9)
	Unknown		36 (10.4)
Maternal employment, No. (%)		351	
	Unemployed or social support		47 (13.4)
	Working part time		90 (25.6)
	Working full time		67 (19.1)
	Homemaker		119 (33.9)
	Other		7 (2)
	Unknown		21 (6)
Paternal employment, No. (%)		349	
	Unemployed or social support		18 (5.2)
	Working part time		18 (5.2)
	Working full time		259 (74.2)
	Homemaker		5 (1.4)
	Other		11 (3.2)
	Unknown		38 (10.9)
Adults in household, No. (%)		338	
	1		45 (13.3)
	2		267 (79)
	>2		26 (7.7)
Siblings in family, No. (%)		335	
	None		90 (26.9)
	1		158 (47.2)
	>1		87 (26)

ADI-R, Autism Diagnostic Interview–Revised; ADOS, Autism Diagnostic Observation Schedule; ICD-10, International Statistical Classification of Diseases and Related Health Problems, Tenth Revision; KABC, Kaufman Assessment Battery for Children; PDD, pervasive developmental disorder unspecified. ^1^ Higher scores indicate greater severity (ranges of possible scores: total, 0–37; social affect, 0–27; language and communication, 0–9; reciprocal social interaction, 0–19; restricted and repetitive behavior, 0–10). ADOS scores were adapted as described in [32]. ^2^ Higher scores indicate greater severity (ranges of possible cores: reciprocal social interaction, 0–30; language and communication, 0–26; repetitive behaviors and interests, 0–12; early onset, 0–5). ^3^ IQ was assessed with standardized scales. If the child was unable to complete a standardized test, no quantitative IQ assessment was made, but only a categorical clinical judgment whether intellectual disability (ie, IQ < 70) was present. ^4^ Higher scores indicate greater cognitive ability. Scores around 100 indicate normal intelligence; scores below 70 indicate intellectual disability.

**Table 2:** Services received by 357 children with ASD.

Service Type	N (%)	Number of Sessions ^1^, M (SD)	Total Number of Hours ^1^, M (SD)
Interventions			
Specialist autism services ^2^	122 (34)	13.39 (15.55)	47.24 (91.14)
Sensory/motor therapy	149 (42)	13.01 (10.62)	9.16 (8.28)
Speech/language therapy	205 (57)	11.35 (9.04)	7.29 (5.23)
Play therapy	35 (10)	12.71 (10.28)	8.78 (5.47)
Behavioural intervention	55 (15)	24.2 (17.4)	33.11 (35.82)
Social skills training	31 (9)	9.42 (6.65)	8.88 (9.62)
Therapeutic recreational activities	47 (13)	9.3 (7.15)	11.23 (27.06)
Other services (see text)	59 (17)	11.39 (12.31)	11.88 (20.29)
Schooling			
Attends school	222 (62)		
Full-time child care (7 h or more per day)	80 (22)		
Part-time child care (Less than 7 h per day)	38 (11)		
None of the above	17 (5)		
Other services			
Institutional stay ^3^	12 (3)	1.42 (0.67)	1.58 (1.38)
Outpatient services ^4^	37 (10)	1.22 (0.75)	
Medication ^5^	109 (31)		
Diet	59 (17)		
Caregiver time investment			
Caregiver time spent on services (hours/week)	175 (49)	8.74 (13.24)	
Reduction in working hours (percent of full-time)	191 (54)	63.85 (34.19)	
Child care help	47 (13)		
Additional aide at school or home (hours/week)	108 (30)	19.16 (11.04)	

All data represent 2 months before the baseline. Total *n* = 357 (excluding 7 with missing quality of life scores at baseline). ^1^ Of those who received this service. ^2^ Specialist autism services includes special education schools specifically for children with ASD (German: Frühförderung; Norwegian: autismeteam). ^3^ Number of stays, total number of days. ^4^ Number of visits. ^5^ Number of days. SD, Standard Deviation; M, Median.

**Table 3:** Health services and societal costs received 357 children with ASD.

Service Type	M (SD)	Range
Health services costs		
Specialist autism services	314.91 (976.21)	0 to 6048
Sensory/motor therapy	213.18 (459.5)	0 to 3360
Speech/language therapy	251.42 (405.33)	0 to 2016
Play therapy	31.54 (194.23)	0 to 2184
Behavioural intervention	283.64 (1363.25)	0 to 16,800
Social skills training	12.36 (63.85)	0 to 672
Therapeutic recreational activities	22.53 (178.11)	0 to 3024
Other services	62.69 (347.51)	0 to 4200
Institutional stay	2.75 (51.9)	0 to 980.61
Outpatient services	1.18 (10.23)	0 to 131.64
Medication	6.17 (29.45)	0 to 289.62
Diet	7.22 (35.49)	0 to 455.14
Sum of all health services costs	1209.59 (2159.84)	0 to 24,780
Societal costs		
Childcare/school	460.59 (160.32)	0 to 707.57
Reduction in working hours	523.86 (642.82)	0 to 1946.08
Child care help (private)	18.24 (124.99)	0 to 1645.54
Additional aide at school or home	621.28 (1145.96)	0 to 4288
Sum of all societal costs	1623.96 (1316.7)	0 to 6263.26
Sum of all costs	2833.56 (2497.5)	84 to 26,050.54

SD, Standard Deviation; M, Median.

**Table 4:** Predictors of costs: All participants.

Source	Health Costs (All Participants)		Health Costs (Outlier Excluded)		Societal Costs (All Participants)	
	b (SE)	*p*	b (SE)	*p*	b (SE)	*p*
Age (continuous)	207.63 (133.38)	0.120	213.20 (107.64)	0.048 *	32.99 (79.46)	0.678
Severity (continuous)	14.74 (23.55)	0.532	−15.47 (19.13)	0.419	−16.68 (14.03)	0.235
Intellectual disability (binary)	320.40 (249.90)	0.201	566.11 (202.48)	0.005 **	−71.95 (148.88)	0.629

b, beta weight; SE, standard error; * *p* < 0.05; ** *p* < 0.01.

**Table 5:** Services received by 217 children with ASD in Europe.

Service Type	*n* (%)	Number of Sessions ^1^, M (SD)	Total Number of Hours ^1^, M (SD)
Interventions			
Specialist autism services ^2^	50 (14)	7.48 (5.04)	14.94 (16.76)
Sensory/motor therapy	72 (20)	10 (7.13)	7.92 (5.38)
Speech/language therapy	109 (31)	8.72 (5.31)	6.16 (4.11)
Play therapy	21 (6)	15.52 (12.29)	10.63 (6.04)
Behavioural intervention	19 (5)	26.53 (22.14)	35.61 (34.62)
Social skills training	15 (4)	12.6 (7.73)	9.84 (9.98)
Therapeutic recreational activities	25 (7)	7.64 (4.68)	4.8 (2.62)
Other services	25 (7)	11.88 (12.3)	14.47 (28.14)
Schooling			
Attends school	121 (34)		
Full-time child care (7 h or more per day)	65 (18)		
Part-time child care (Less than 7 h per day)	26 (7)		
None of the above	5 (1)		
Other services			
Institutional stay ^3^	10 (3)	1.5 (0.71)	1.8 (1.4)
Outpatient services ^4^	24 (7)	1.12 (0.45)	
Medication ^5^	55 (15)		
Diet	38 (11)		
Caregiver time investment			
Caregiver time spent on services (hours/week)	85 (24)	6.54 (14.84)	
Reduction in working hours (percent of full-time)	111 (31)	65.37 (32.24)	
Child care help	26 (7)		
Additional aide at school or home (hours/week)	77 (22)	19.81 (10.59)	

All data represent 2 months before the baseline. ^1^ Of those who received this service. ^2^ Specialist autism services includes special education schools specifically for children with ASD (German: Frühförderung; Norwegian: autismeteam). ^3^ Number of stays, total number of days. ^4^ Number of visits. ^5^ Number of days. SD, Standard Deviation; M, Mean.

**Table 6:** Health services and societal costs received by 217 children with ASD in Europe.

Service Type	M (SD)	Range
Health services costs		
Specialist autism services	98.29 (244.31)	0 to 1512
Sensory/motor therapy	122.28 (283.22)	0 to 1960
Speech/language therapy	200.77 (337.66)	0 to 2016
Play therapy	48.39 (247.19)	0 to 2184
Behavioural intervention	186.79 (1102.81)	0 to 13,440
Social skills training	13.8 (74.46)	0 to 672
Therapeutic recreational activities	7.19 (35.73)	0 to 336
Other services	53.01 (252)	0 to 2436
Institutional stay	0 (0)	0 to 0
Outpatient services	0.28 (4.12)	0 to 60.69
Medication	1.73 (16.12)	0 to 228.97
Diet	7.68 (38.99)	0 to 455.14
Sum of all health services costs	740.19 (1322.7)	0 to 14,042
Societal costs		
Childcare/school	491.63 (155.58)	0 to 707.57
Reduction in working hours	486.91 (589.6)	0 to 1946.08
Child care help (private)	16.53 (110.73)	0 to 1320.9
Additional aide at school or home	753.36 (1220.87)	0 to 4288
Sum of all societal costs	1748.43 (1344.59)	0 to 6263.26
Sum of all costs	2488.62 (1951.48)	101.14 to 15,856.9

SD, Standard Deviation; M, Mean.

## Data Availability

Deidentified individual participant data for this study are available in the Norwegian Centrefor Research Data (NSD) public repository at http://dx.doi.org/10.18712/NSD-NSD2466-V1 (accessed on 5 March 2021).
